# Congenital Mid Ureteric Valve Stenosis Revisited: Case Report and Review of the Literature

**DOI:** 10.3389/fped.2019.00108

**Published:** 2019-03-29

**Authors:** Mohammed Elifranji, Abderrahman Elkadahi, Adrian Charles, Tariq O. Abbas

**Affiliations:** ^1^Department of Pediatric Surgery, Hamad General Hospital, Doha, Qatar; ^2^Pathology Department, Sidra Medicine, Doha, Qatar; ^3^College of Medicine, Qatar University, Doha, Qatar; ^4^Surgery Department, Weil Cornell Medicine-Qatar, Doha, Qatar

**Keywords:** congenital, PUJ obstruction, valve, hydronephrosis, ureter, retrograde pyelography

## Abstract

Congenital mid ureteric valve (MUV) stenosis is a very rare entity. Definitive preoperative diagnosis is clinically challenging, and most patients are misdiagnosed preoperatively. Intraoperative identification is therefore very important. Curative treatment consists of excision of the involved ureteric segment and anastomosis. This report describes the clinical findings in a patient with congenital mid ureteric valve stenosis, including radiological and histological workup and operative management. Routine intraoperative retrograde pyelography is important in the diagnosis of such rare pathologies.

## Introduction

Congenital mid ureteric valve (MUV) stenosis is a very rare cause of ureteric obstruction and hydronephrosis (HN) in children. Since initially described in 1877, only about 65 patients have been diagnosed with congenital MUV stenosis (1). Most children who present with this condition are initially diagnosed with more common conditions, including pelviureteric junction (PUJ) obstruction and megaureter (MU) (2). Therefore, a high level of suspicion is required. This report describes the clinical findings in a patient with congenital MUV stenosis, including radiological and histological workup, and operative management.

## Case Report

**Figure 1:**
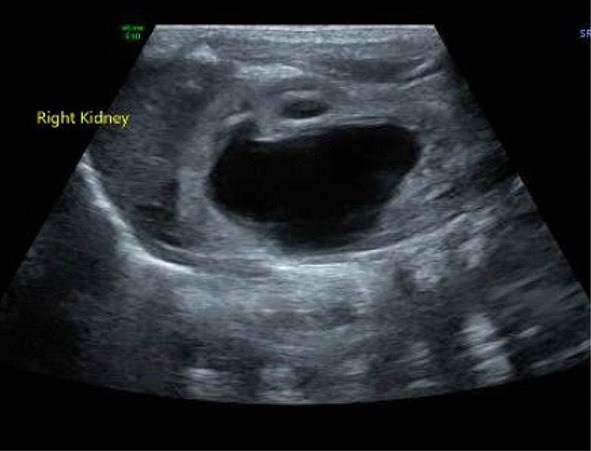
Ultrasound of the right kidney, demonstrating significant hydronephrosis with calyceal blunting and non-visualization of the right proximal ureter.

**Figure 2:**
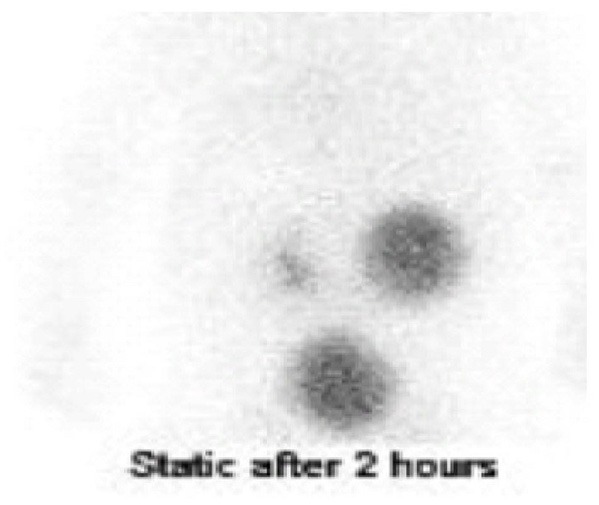
Diuretic renal scintigraphy with Tc 99m DTPA. Delayed images showed tracer retention by the hydronephrotic right kidney, with normal excretion by the left kidney. In addition, the proximal right ureter was dilated. The relative renal split function was 40% for the right kidney and 60% for the left kidney.

The patient was a 4-month-old boy born at gestational age of 36 weeks by elective cesarean section because of placenta previa. During the third trimester, he was found to have right hydronephrosis, with an anteroposterior diameter (APD) of 27 mm and SFU 4. His left kidney and urinary bladder were normal, as were his initial physical examination and laboratory workup at birth. A voiding cystourethrogram at 1 day of age showed a normal bladder and urethra and no evidence of vesicoureteric reflux. Ultrasound examination showed right hydronephrosis with an APD of 26 mm (Figure 1). Diuretic renal scintigraphy with Tc 99m DTPA showed right renal pelvic dilatation with an obstructive pattern of radiotracer washout and a differential renal function of 40% (Figure 2). Follow-up renal ultrasound at 2 months of age showed the persistence of high grade right hydronephrosis with mild thinning of the renal cortex.

**Figure 3:**
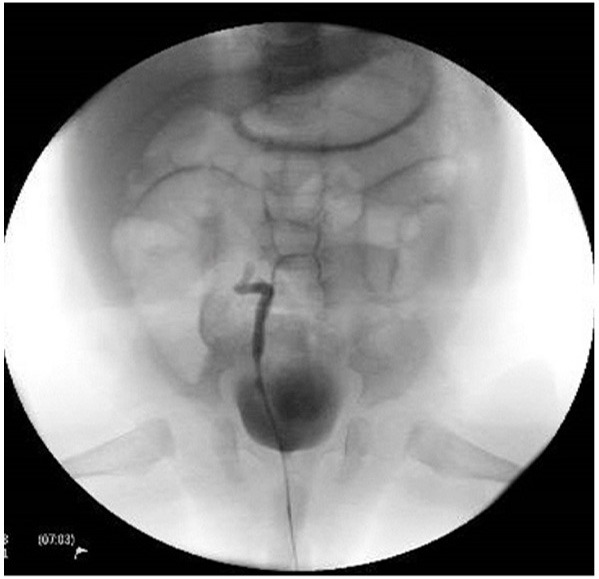
Retrograde pyelography showing non-visualization of the right upper ureter with contrast pushed to the bladder upon application of higher pressure.

Based on a preoperative working diagnosis of right pelviureteric junction obstruction, the patient was scheduled for right pyeloplasty. Routine intraoperative cystoscopy and right retrograde pyelography prior to pyeloplasty showed that the contrast was unable to pass beyond a proximal ureteric narrowing, with subsequent application of higher pressure resulting in reflux toward the urinary bladder (Figure 3).

**Figure 4:**
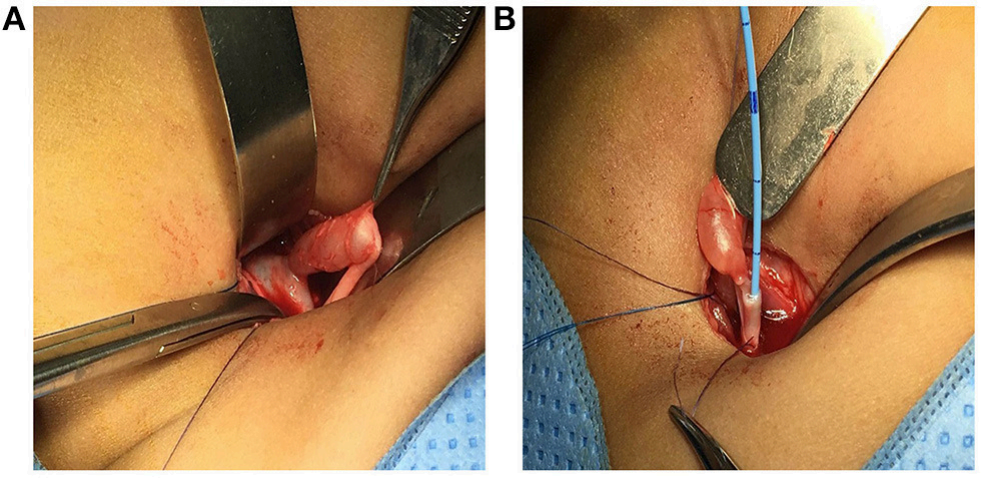
Intra operative images in this patient. **(A)** A patent and wide pelviureteric junction (PUJ) was observed, along with a dilated right upper ureter with a demarcation point about 4 cm from the PUJ. **(B)** A transverse 2 mm ureterotomy just distal to the size discrepancy point, showing no free flow of urine.

**Figure 5:**
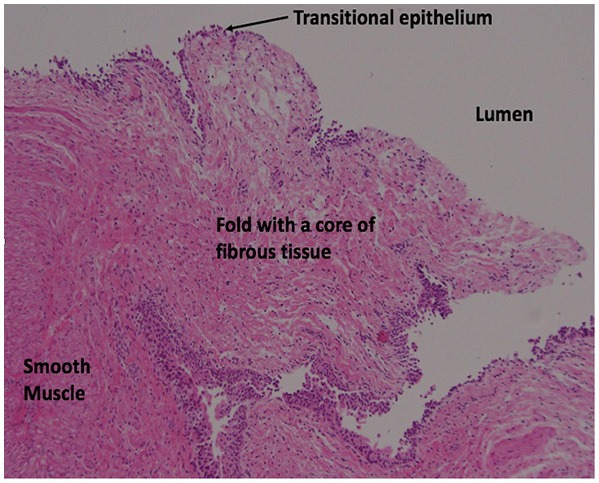
Histological evaluation of one side of the excised ureteric segment, showing a tongue-like fibrous fold derived from smooth muscle cells and extending toward the lumen (right upper side) of the ureter with a relative absence of epithelial lining (hematoxylin and eosin staining, x 10).

Surgery began with a transverse muscle splitting incision through the right upper abdomen. The right pelvic-ureteric junction was wide and patent (Figure 4A). However, right ureteric focal narrowing was observed 4 cm from the pelviureteric junction, with distal ureterotomy showing no flow of urine through the right ureter (Figure 4B). This short segment, about 3 mm in length, was opened longitudinally in a retrograde manner, revealing a diaphragm-like transverse valve. This ureteric segment was excised and an end-to-end ureteroureterostomy was fashioned following spatulation of the two ends over a DJ stent, which was removed uneventfully after 5 weeks. Histological examination of the excised ureteric segment showed normal urothelial lining, with no evidence of fibrosis or inflammation, and a valve-like membranous protrusion perpendicular to the wall of the ureter, which did not contain smooth muscles or an extensive urothelial covering (Figure 5). The patient remained asymptomatic on follow-up, with ultrasound imaging 3 months later showing improvement of right renal hydronephrosis.

## Discussion

Although congenital narrowing at either end of the ureter is uncommon, congenital MUV stenosis is considered rare. Although its cause is unclear, several pathogenic hypotheses have been proposed, including abnormal ureteric recanalization (3), intrauterine ureteritis (4), and extrinsic vascular compression.(5)

Congenital mid ureteric narrowing may be due to strictures and ureteral valves. Mid ureteric strictures have been attributed to ischemic events during embryonic development of the ureter. These strictures have been observed where the ureter crosses the internal iliac arteries, a region considered a vascular watershed transition area, as the ureteral blood supply at that point changes from the abdominal aorta to the iliac and hypogastric arteries (6). Strictures can be caused by abnormal or disorganized arrangements of the ureteral wall musculature, with or without fibrosis, accompanied by a normal urothelium. However, in severe strictures, the smooth muscle layer is replaced by fibrous tissue. Moreover, these conditions have high rates of associations with other urinary tract abnormalities (7, 8).

Histological criteria for the diagnosis of ureteral valve strictures include demonstrable transverse folds of the ureteral mucosa containing bundles of smooth muscle fibers, obstructive changes proximal to the valve with a normal ureter distally, and no other evidence of mechanical or functional obstruction (9). A later study reported that a true valve can lack smooth muscle in the fold itself, as long as smooth muscle fibers are present at the base of the valve, the urothelium is normal and the other criteria are met (10). Findings in the present patient support the last definition.

Routine retrograde pyelography is recommended to avoid missing unexpected intraoperative findings, as in our patient. Alternatively, preoperative magnetic resonance urography (MRU) can accurately delineate anatomical abnormalities and properly diagnose these patients (11).

The treatment of choice for congenital mid ureter stenosis consists of excision of the affected ureteric segment and anastomosis (12, 13). Various minimal invasive techniques including laparoscopic resection and uretero-ureterostomy have been described in the literature (14). Endosurgical procedures, such as endoscopic incision of the stricture or dilatation, may be considered although they have low success rates (7).

In summary, congenital MUV stenosis is a rare but important cause of hydronephrosis. Pre-operative detection of this condition is clinically challenging and requires a high level of attention. Intraoperative cystoscopy plus retrograde pyelography are recommended, especially when pre-operative images are equivocal. Tension-free primary ureteroureterostomy is the most effective treatment option.

## Data Availability

All datasets generated for this study are included in the manuscript and/or the Supplementary Files.

## Ethics Statement

The project has been approved by Medical Research Center (MRC) of Hamad Medical Corporation (HMC). Study Number (MRC 04-18-309).

## Consent for Publication

Written informed consent was obtained from the parent of the patient for publication of this case report.

## Author Contributions

ME prepared and wrote the article. AC evaluated the pathology specimen. AE and TA have reviewed and critically analyzed the manuscript.

### Conflict of Interest Statement

The authors declare that the research was conducted in the absence of any commercial or financial relationships that could be construed as a potential conflict of interest.
